# Internal Mammary Artery Injury without Chest Wall Fractures after Cardiopulmonary Resuscitation: A Case Report

**DOI:** 10.1155/2018/1948151

**Published:** 2018-10-23

**Authors:** Sayuri Tokioka, Shinichiro Masuda, Masamitsu Shirokawa, Takashi Shibui

**Affiliations:** ^1^Department of Cardiology, Tokyo Metropolitan Hiroo Hospital, 10-34-2, Ebisu, Shibuya-ku, Tokyo 153-0013, Japan; ^2^Emergency Care Center, Tokyo Metropolitan Hiroo Hospital, 10-34-2, Ebisu, Shibuya-ku, Tokyo 153-0013, Japan

## Abstract

High-quality cardiopulmonary resuscitation (CPR) is crucial for survival from cardiac arrest. However, various chest compression-associated injuries have been reported. Internal mammary artery (IMA) injury is one of the rare complications after CPR, and most of cases include rib and sternum fractures. In this report, we describe a rare case of IMA injury without chest wall fractures after CPR. An 85-year-old man with a history of acute myocardial infarction 2 weeks prior visited to our hospital for sustained ventricular tachycardia (VT). After admission, sustained VT requiring CPR occurred several times. Emergency coronary angiogram revealed 90% stenosis at the left anterior descending artery. Hence, emergency percutaneous coronary intervention (PCI) was performed. During the PCI, blood gas analysis showed decreasing serum hemoglobin levels. Contrast computed tomography revealed hemothorax and extravasation at the branch of the right IMA without chest wall fractures. The patient's deteriorating hemodynamic condition precluded thoracotomy or embolization to stop the bleeding. The patient died on the next day of hospitalization. IMA injury can occur after CPR, regardless of chest wall fractures and can be fatal without early diagnosis. For an emergency physician, IMA injury should be considered as a cause of unknown anemia after CPR.

## 1. Introduction

High-quality cardiopulmonary resuscitation (CPR) is crucial for survival from cardiac arrest [1]; however, various chest compression-associated injuries are reported [2]. Although rare, internal mammary artery (IMA) injury after CPR can be fatal [3]. The precise mechanism of IMA injury has not been established, but a possible cause is fracture of the ribs or sternum, which often occurs after CPR. Herein, we report a case of IMA injury without chest wall fractures after CPR that was diagnosed by computed tomography (CT).

## 2. Case Presentation

**Figure 1:**
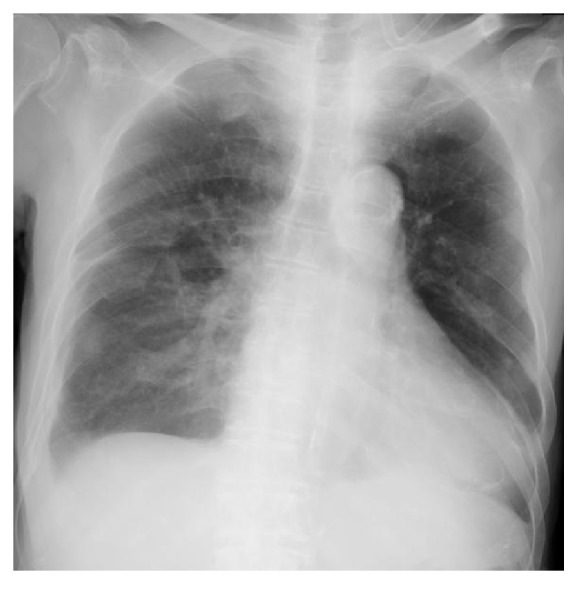
Chest radiography on admission revealed mild widening of cardiac silhouette and right pleural effusion.

**Figure 2:**
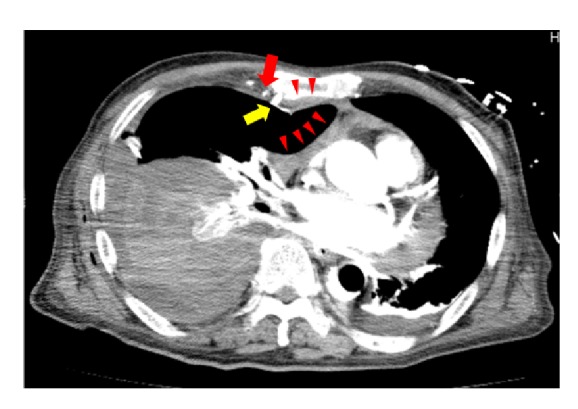
Contrast computed tomography (CT) scan on the next day of hospitalization. Right internal mammary artery (IMA) is divided into branches. A red arrow shows main branch of IMA and a yellow arrow shows the extravasation at a branch of IMA. There is small anterior mediastinal hematoma (red arrowheads) around the extravasation. Massive bloody pleural effusion is confirmed in the right lung.

**Figure 3:**
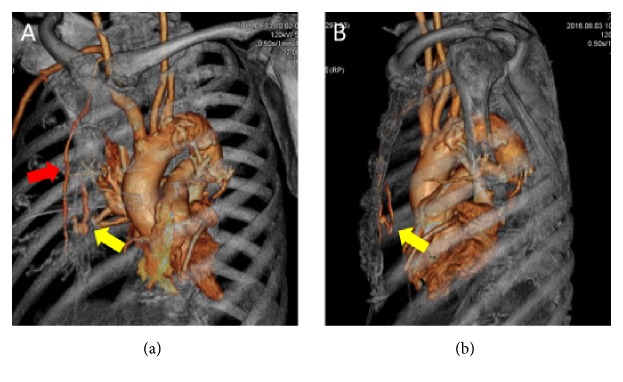
Three-dimensional contrast CT images. Left posteroanterior oblique view (a) and left-right view (b). The extravasation from a branch of IMA (yellow arrow) is confirmed. A red arrow shows the IMA main branch.

An 85-year-old man visited our hospital for treatment of sustained ventricular tachycardia (VT), which caused an unstable hemodynamic state. His medical history was significant for acute myocardial infarction (AMI) 2 weeks prior, and a drug-eluting stent was implanted in the left main trunk. On admission, the patient was asymptomatic. His vital signs were as follows: blood pressure, 144/78 mm Hg; pulse rate, 80 beats/min; respiratory rate, 16 breaths/min. His oxygen saturation was 99% in room air, whereas his laboratory test showed the following results: sodium, 140 mmol/L; potassium, 4.5 mmol/L; creatine kinase, 28 U/L; creatine kinase-muscle/brain, 9 U/L. Electrocardiography showed sinus rhythm with T-wave inversions in the I, aV_L_, and V1-V6 leads. Chest radiography revealed mild widening of the cardiac silhouette and right pleural effusion (Figure 1). After the admission, he presented with sustained VT. Intravenous amiodarone was administered and CPR was performed, but amiodarone was ineffective in terminating VT. Hence, emergency coronary angiography (CAG) was performed for suspected myocardial ischemia. Intra-aortic balloon pumping and percutaneous cardiopulmonary support were established before emergency CAG because of his unstable hemodynamic state. CAG revealed 90% stenosis at the middle segment of the left descending artery, thus, ad hoc percutaneous coronary intervention (PCI) was performed. During the PCI, his serum hemoglobin level decreased from 11.1 to 3.0 g/dL. Chest radiography revealed pleural effusion in the right lung, which was not present on admission. Noncontrast CT was performed after emergency PCI, which revealed right hemothorax without any chest wall fractures. A right chest tube was placed, and 1.8 L of pleural effusion was drained over 6 hours. Despite blood transfusion of 12 units of red blood cells and 12 units of fresh frozen plasma, his serum hemoglobin level decreased, suggesting persistent blood loss. To identify the source of the bleeding, contrast CT was performed, which revealed small anterior mediastinal hematoma and bloody pleural effusion (Figure 2). Three-dimensional contrast CT demonstrated an extravasation at the branch of the right IMA (Figures 3(a) and 3(b)). The patient's deteriorating hemodynamic condition precluded thoracotomy or embolization to stop the bleeding. He subsequently died on the next day of hospitalization.

## 3. Discussion

This case demonstrated that rare case of IMA injury related to CPR without chest wall fractures. IMA injury after CPR can be a life-threatening complication as it results in severe hypovolemic shock, ongoing blood loss, and extrapleural hematoma [3]. The association between chest wall fractures and IMA injury after CPR has not been well investigated; however, most of previous clinical cases presenting IMA injury after CPR often occurred at the level of the middle IMA and include rib and sternum fractures, which are thought to injure the IMA directly [3–7]. In the present case, chest wall fractures were not confirmed. The possible mechanism of this case was shear stress, which is known to be a mechanism of blunt traumatic vascular injury [8, 9]. Shear stress generates boundary layer separation; short and sudden compression causes a sudden decrease in vessel diameter and changes in blood flow direction, with fluid near the wall reversing the direction of the main flow [10]. In the present case, IMA injury occurred at the middle level without chest wall fractures. It is estimated that, at middle level of IMA, the force of chest compression is directly transmitted and shear stress occurs easily.

Contrast CT has been reported as a useful modality for detecting the source of bleeding and can help facilitate treatment with thoracotomy and transcatheter arterial embolization [11]. The blood flow rate in the IMA is 150 mL/min, and it has many branches to the mediastinum and chest wall [12]. Hence, IMA injury is likely to present rapid deterioration of the hemodynamic state once it occurs. In the present case, although contrast CT was useful for detecting the IMA injury, surgical treatment or transcatheter arterial embolization could not be performed because the hemodynamic state deteriorated rapidly. Delayed diagnosis resulted in fatal outcome because IMA injury was not considered as a differential diagnosis in this scenario.

In conclusion, IMA injury after CPR should be considered as a differential diagnosis in a patient presenting with bleeding complications despite the absence of chest wall fractures. Contrast CT is strongly recommended as soon as possible in all surviving patients after CPR to avoid fatal outcome.
